# Different narcotic gases and concentrations for immobilization of ostrich embryos for *in-ovo* imaging

**DOI:** 10.3389/ebm.2024.10037

**Published:** 2024-05-24

**Authors:** O. Perkas, A. Schmidt, C. Kuehnel, J. Greiser, H. Hermeyer, C. Klingner, M. Freesmeyer, T. Winkens

**Affiliations:** ^1^ Clinic of Nuclear Medicine, Jena University Hospital, Jena, Thuringia, Germany; ^2^ Translational Nuclear Medicine and Radiopharmacy, Clinic of Nuclear Medicine, Jena University Hospital, Jena, Thuringia, Germany; ^3^ Department of Neurology, Jena University Hospital, Jena, Germany; ^4^ Biomagnetic Center, Jena University Hospital, Jena, Germany

**Keywords:** in-ovo imaging, ostrich eggs, animal model, biomagnetism, alternative animal testing, narcotic gases, magnet-ovography

## Abstract

*In-ovo* imaging using avian eggs has been described as a potential alternative to animal testing using rodents. However, imaging studies are hampered by embryonal motion producing artifacts. This study aims at systematically comparing isoflurane, desflurane and sevoflurane in three different concentrations in ostrich embryos. Biomagnetic signals of ostrich embryos were recorded analyzing cardiac action and motion. Ten groups comprising eight ostrich embryos each were investigated: Control, isoflurane (2%, 4%, and 6%), desflurane (6%, 12%, and 18%) and sevoflurane (3%, 5%, and 8%). Each ostrich egg was exposed to the same narcotic gas and concentration on development day (DD) 31 and 34. Narcotic gas exposure was upheld for 90 min and embryos were monitored for additional 75 min. Toxicity was evaluated by verifying embryo viability 24 h after the experiments. Initial heart rate of mean 148 beats/min (DD 31) and 136 beats/min (DD 34) decreased over time by 44–48 beats/minute. No significant differences were observed between groups. All narcotic gases led to distinct movement reduction after mean 8 min. Embryos exposed to desflurane 6% showed residual movements. Isoflurane 6% and sevoflurane 8% produced motion-free time intervals of mean 70 min after discontinuation of narcotic gas exposure. Only one embryo death occurred after narcotic gas exposure with desflurane 6%. This study shows that isoflurane, desflurane and sevoflurane are suitable for ostrich embryo immobilization, which is a prerequisite for motion-artifact free imaging. Application of isoflurane 6% and sevoflurane 8% is a) safe as no embryonal deaths occurred after exposure and b) effective as immobilization was observed for approx. 70 min after the end of narcotic gas exposure. These results should be interpreted with caution regarding transferability to other avian species as differences in embryo size and incubation duration exist.

## Impact statement

In-ovo imaging represents an adequate alternative for preclinical imaging sparing animal research using rodents. In order to avoid image artifacts caused by embryonal motion, use of isoflurane has been described previously. This work systematically investigated different narcotic gases and concentrations, showing successful immobilization for more than one hour after exposure using highest concentrations of isoflurane, desflurane and sevoflurane. This information is needed for planning and execution of in-ovo imaging experiments.

## Introduction

*In-ovo* imaging has been described as a potential alternative concept to animal testing using rats or mice [1–4]. According to national and international legislation, research using eggs does not qualify as animal testing as long as all experiments are carried out before hatching [5–8].

*In-ovo* methods comprise experiments using chorio-allantois membrane (CAM), vaccine research and production, toxicity studies as well as cardiovascular research, just to name a few. The largest research area is covered by CAM assays, because this highly vascularized membrane enables basic research regarding cancer (tumor cell growth [9], xenografts [10], epithelial-mesenchymal transition [11], circulating tumor cells [12]), angiogenesis (pro- and antiangiogenetic drugs) [13, 14], wound healing, stem cells [15] and even serves as training platform for surgical procedures [16]. As these models have been shown to provide essential information about cell interactions, cancer and drug effects [17], they are suitable to answer important questions of basic research [18, 19]. Regarding the role of *in-ovo* methods within the landscape of research in general, drug development is chosen as an example: It is commonly accepted that new drugs are tested on animals (i.e., rodents) before first application in humans in order to sort out drug candidates which are associated with toxicities. If there was a tool that allowed for an even earlier selection of promising drug candidates, this would reduce the number of animals needed.

With regard to preclinical imaging, *in-ovo* methods contribute to reduction of classic animal research using rodents as it can serve as a “pre-selection tool” in order to test and dismiss unfavorable experimental approaches (e.g., radiopharmaceutical substances for nuclear medicine imaging), so only promising experimental approaches are tested on rodents. Thus, *in-ovo* research complies with the principles of modern animal testing (3R) as established by Russel and Burch in 1959 [20].

Usually, chicken embryos are used for *in-ovo* imaging, however, this requires dedicated small animal imaging devices which represents a disadvantage regarding limited access [1, 2, 4]. A concept using substantially larger ostrich eggs and imaging devices commonly used in routine clinical examinations in humans (e.g., computed tomography, CT; magnet resonance imaging, MRI; and positron emission tomography, PET) has been published before [1, 2, 4].

Preclinical *in-vivo* imaging requires immobilization in order to produce artifact-free imaging [21, 22]. In rodents, isoflurane anesthesia is an established method [23]. Previous publications report on the effect of isoflurane on chicken and ostrich embryos and have investigated feasibility and success of reduction of embryonal movements [1, 22]. However, there are no studies that explore the effect of different narcotic gases and different concentrations.

Thus, this study aims at comparing isoflurane, desflurane and sevoflurane in three different concentrations. The frequency of movements and heart rate is assessed using biomagnetic signals. Furthermore, embryo survival after narcotic gas exposure is evaluated. Two different development stages of ostrich embryos are investigated, i.e., development day (DD) 31 and 34.

## Materials and methods

### Ostrich eggs

Ostrich eggs were obtained from a local ostrich farm 15 km from the research facility between April and September. Artificial incubation was started 1–4 days after laying and was carried out using a multistage egg incubator (Sofie 3, Hemel, Verl, and Germany) with constant incubation properties at 36.5°C and 25% air humidity as described elsewhere [2, 4]. The ostrich eggs used in this study were part of a larger research project comprising different experiments. During the time period, a total of 373 ostrich eggs were obtained. Non-fertilized eggs and eggs containing dead embryos were discarded. After 31 days of incubation, 188 ostrich embryos were available for different experiments and of these, 85 ostrich embryos were randomly chosen to be investigated with different narcotic gases as described in this study. Initial egg weight was 1,398 ± 112 g.

If artificially incubated, ostrich eggs usually hatch after 42 days [24]. As it was a requirement to end all experiments before hatching, studies were performed on DD 31 and DD 34. This embryo study did not qualify as an animal research study according to the Federal German Animal Protection Act. Registration took place with the Office for Consumer Protection of the Thuringia State, registration number 22-2684-04-02-114/16. Thus, ethic committee’s approval was waived.

### Magnet-ovography (MOG)

Methodological aspects of detecting embryonal movement and cardiac action using standard magnetencephalograph (MEG, Neuromag, Elekta, Sweden) systems have been published before [1]. In short, MEG consists of multiple magnetometers, measuring magnetic flux and background noise [25]. Each magnetometer produces a graph (channel) showing the magnetic field change over time. Due to the negative influence of ferromagnetic objects close to the investigation site, all components have to be designed metal-free and thus, remote controls consisting of tubes and a chamber to hold the ostrich egg were used to apply different narcotic gases as described before [1].

### Data acquisition and post-processing

Data acquisition followed chronological steps as outlined by Freesmeyer et al. comprising optimal egg positioning via visual verification of heart signals, followed by automated signal recording of each magnetometer channel [1]. Regarding data post-processing, cardiac signals were detected using a method which has been validated for human fetus observations described by Schmidt et al. in 2019, analyzing information about the signal amplitude (minimum-maximum), the overall signal strength, signal space angle and moving correlation coefficient [26]. Movement signals were evaluated by methods of Schmidt et al. (i.e., advanced automatic movement detection, using position changes of the heart signal vector over time) and Freesmeyer et al. (i.e., manual movement detection, using a threshold-based analysis of different signal frequency-bands of each magnetograph channel) [1, 26]. In order to ensure comparability for both methods, a data set comprising two groups (control, isoflurane 6%) from Freesmeyer et al. was analyzed using both methods and agreement was determined using Bland-Altmann plots. The individual embryos studied by Freesmeyer et al. were the same as investigated in this analysis [1]. The other groups (isoflurane 4%, isoflurane 2%, desflurane, sevoflurane) were evaluated only using advanced automatic movement detection method due to time aspects. This schedule was deemed adequate in view of time efficiency as estimated duration for manual movement detection (Freesmeyer et al.) [1] is longer than advanced automatic movement detection (Schmidt et al.) [26].

### Experimental design

Isoflurane (Piramal Healthcare, Mumbai, India), Desflurane (Suprane, Baxxter Inc., Illinois, United States) and Sevoflurane (Abbvie Inc., Illinois, United States) were used in three different concentrations according to the available range which was predefined by the respective vaporizers (Vapor 2000 Isofluran, Vapor 2000 Desfluran, Vapor 2000 Sevofluran each by Draeger, Luebeck, Germany). The lowest and highest possible concentration was investigated as well as a concentration in between. Ten groups were investigated: Control, isoflurane (2%, 4%, and 6%), desflurane (6%, 12%, and 18%) and sevoflurane (3%, 5%, and 8%). For each concentration, eight ostrich embryos were investigated on DD 31 and the same ostrich embryos were investigated on DD 34 again. Additionally, a control group comprising 8 ostrich eggs was evaluated without exposure to narcotic gases.

The experiments were conducted as described elsewhere [1]. In short, eggs were removed from the incubator and transferred to MEG facility. Surface temperature was measured using an infrared contactless thermometer (VOLTCRAFT IR 900-30S, Conrad Electronic, Hirschau, Germany) before and after MOG.

**FIGURE 1:**
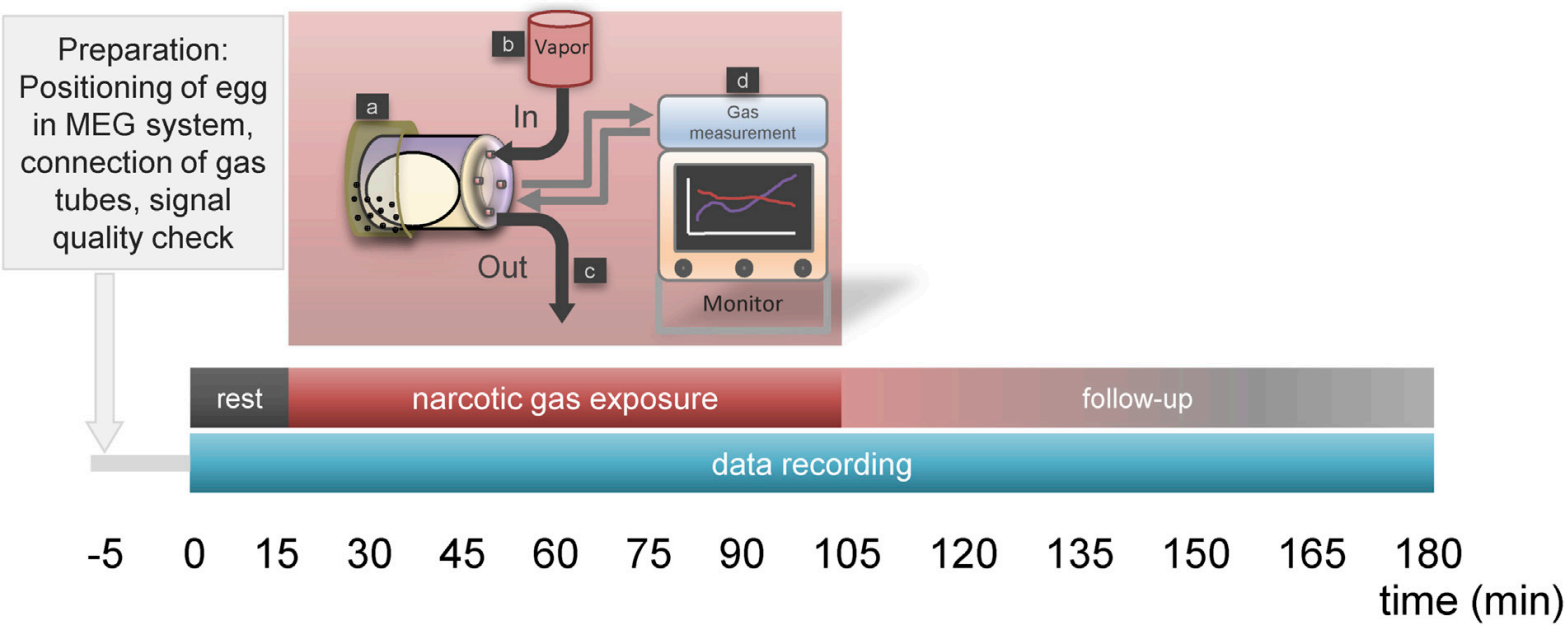
Time schedule for narcotic gas exposure. Phase “rest” (15 min) allows for reduction of embryonal arousal after egg transport to MEG-facility. This phase is followed by “narcotic gas exposure” (90 min) and a “follow-up phase” (75 min). During rest and follow-up phase, ambient air is inflated into the chamber holding the ostrich eggs. During “narcotic gas exposure,” respective narcotic gas is applied. In control group, during this phase ambient air is inflated. During 180 min MOG, the ostrich egg is held by a container which is located at the head rest (a) of the MEG system. Narcotic gas inflow is provided by specific vapor (b) and narcotic gas outflow (c) is provided by exhaustion system. During the whole experiment, narcotic gas concentration was continuously measured using a gas measurement module (d) for anesthetic care (Scio Four Oxi, Draeger, Luebeck, Germany), ensuring adequate concentration of narcotic gas within the chamber holding the ostrich egg.

Eggs were placed into chamber connected to tubes which enabled in- and outflow of narcotic gases and ambient air. The chamber was set into the MEG device and signal quality was visually assessed. If a positive cardiac signal was visible, experiment started according to Figure 1. After a resting phase of 15 min, narcotic gas exposure was started and continuously monitored using a gas measurement module for anesthetic care (Scio Four Oxi, Draeger, Luebeck, Germany). In order to extract the respective narcotic gas from the vapor, ambient air (21% oxygen, 79% nitrogen) was used. The flow was set at 2 L/min and adjusted as needed, always verifying the desired narcotic gas concentration via the gas measurement module. Narcotic gas exposure was upheld for 90 min and then vaporizer was closed. Data acquisition was continued for additional 75 min (follow-up phase) assessing possible re-appearance of embryonal movement. In total, data were continuously acquired for 180 min. During resting phase and follow-up phase, ambient air was inflated into the chamber holding the ostrich egg, preventing accumulation of carbon dioxide and subsequent embryo suffocation. In the control group, ambient air was inflated into the chamber for 180 min at a flow of 2 L/min. After the experiment, eggs were transferred into incubator and viability of ostrich embryos was investigated approx. 24 h later by 5 min MOG measurement assessing for cardiac signals; thus, experiment toxicity was evaluated. After the last experiment (on DD 35; viability assessment), ostrich embryos were subjected to imaging studies on DD 37 after which they were sacrificed on DD 37 at the latest using sodium-pentobarbital. Necropsy comprising organ removal was performed aiming at verification of imaging studies.

### Statistics

Individual embryo heart rate was determined for each minute and median values within each group were calculated and compared. Graphs were designed to visually compare heart rate change over time. ANOVA method was used to determine differences between narcotic gases and concentrations regarding heart rate reduction over time.

Method agreement between manual movement detection and advanced automatic movement detection was evaluated by determination of levels of agreement described by Bland and Altmann [27].

Embryo movement was normalized for individual motion signal level during resting phase. Relative signal change was recorded for each minute and median values within each group were calculated. Groups were compared using graphs. Furthermore, a sigmoid fitting curve was calculated for each experiment and inflection points were determined as the time point of successful immobilization (“sleep”) and re-appearance of movement during follow-up phase (“awake”). Time points were compared for each group using Kruskal-Wallis-Test and *p* = 0.05 as a level of significance.

## Results

In total, 168 MOG comprising 85 ostrich eggs were carried out. Eight data sets from 5 ostrich eggs were excluded from analysis due to erroneous data recording, corrupt data files and inconsistent duration of narcotic gas exposure. One of 80 ostrich embryos died after exposure to desflurane (6%) on DD 34. In 79 ostrich embryos, positive heart signal was obtained approx. 24 h after the last experiment, indicating survival. Necropsy revealed no abnormal findings attributable to narcotic gas exposure.

Between MOG start and end, mean temperature reduction was 6.1°C and different narcotic gases did not differ significantly regarding temperature decrease.

**FIGURE 2:**
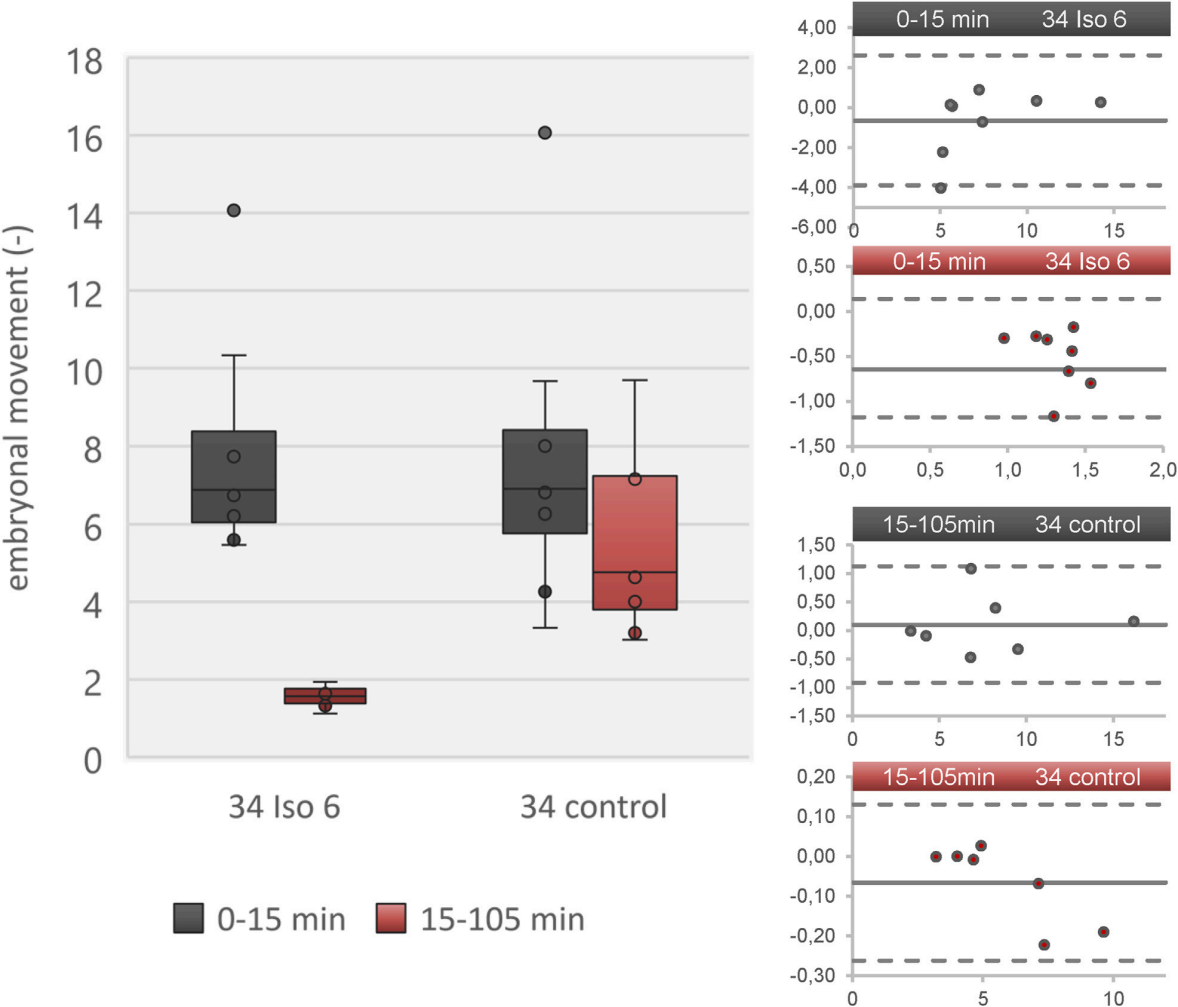
Embryonal movement using advanced automated movement detection according to [26] in a data set derived from [1]. Left: Boxplot diagram showing level of activity during resting phase (0–15 min; grey) and isoflurane/ambient air (15–105 min; red) in two groups (isoflurane 6% and control) on DD 34. Right: Four Bland-Altmann plots showing adequate agreement of advanced automated movement detection and manual movement detection. The four plots refer to the four boxes in the boxplot diagram. The Bland-Altmann plots compare each value between advanced automated movement detection and manual movement detection. The dots indicate the difference between both methods (y-axis) over absolute value (embryonal movement; x-axis) and the solid line represents the mean value of all dots. Dashed lines indicate 2x standard deviation of all differences. All values are in the range of 2x standard deviation, indicating no significant underestimation or overestimation.

Data reconstruction was successful using advanced automatic movement detection method. Comparison with manual movement detection method showed good agreement between control group and isoflurane 6% regarding motion analysis (Figure 2). Duration of data reconstruction and evaluation ranged from 20 min to 160 min per data file comprising 180 min.

### Heart rate

**FIGURE 3:**
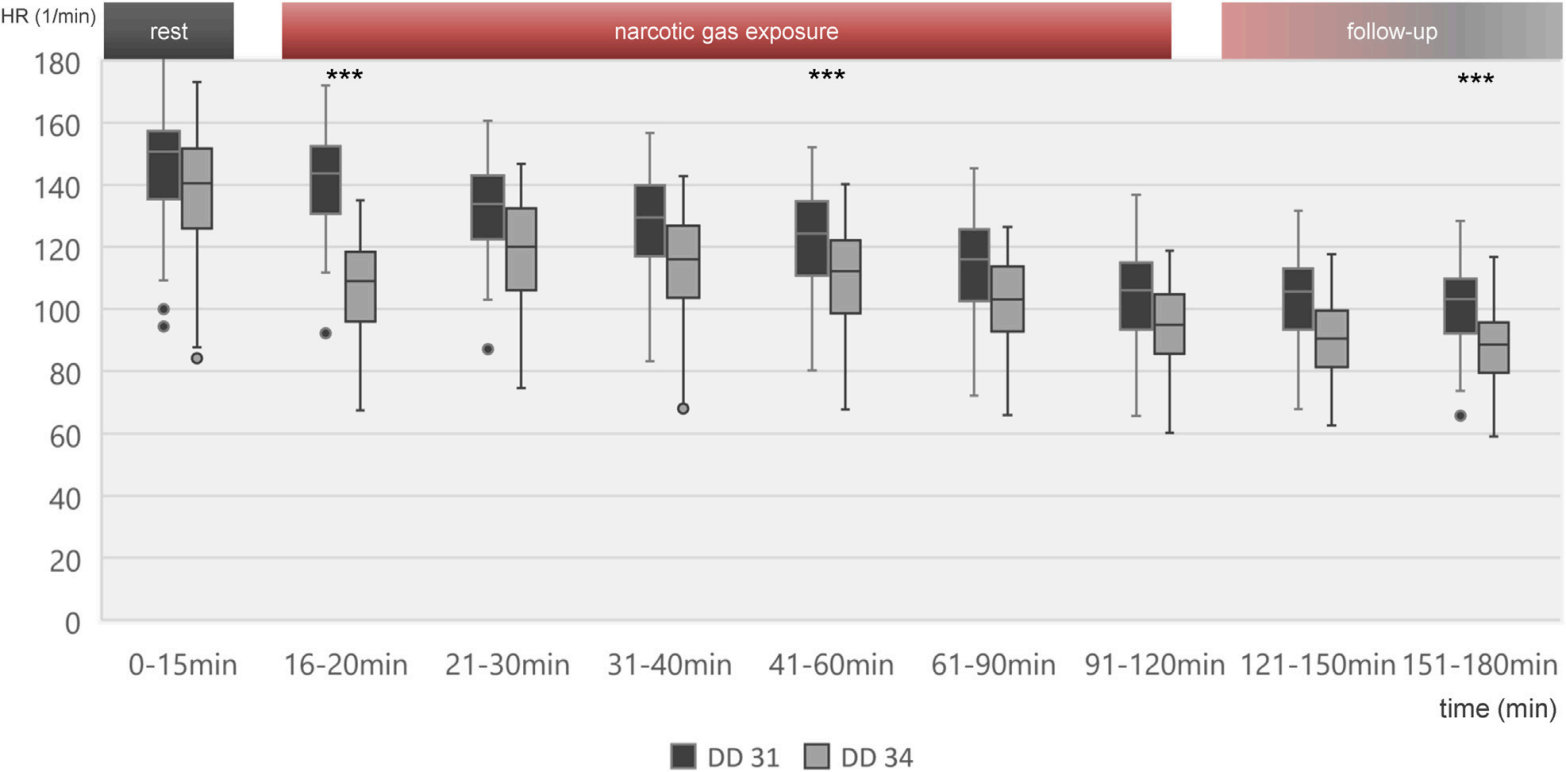
Boxplot diagram of heart rate during different time intervals of all 80 ostrich embryos investigated on DD 31 and 34. Significant differences between DD 31 and DD 34 occur within the first 5 min after initiation of narcotic gas exposure, in the middle of narcotic gas exposure and at the end of follow-up. Aggregated data of all groups including control group was deemed appropriate in view of Figures 4, 5, excluding significant differences between control group and narcotic gases. ***p < 0.001.

Heart rate change over time is shown in Figure 3 for all embryos on DD 31 and DD 34, respectively. On DD 34, ostrich embryos showed significant lower heart rate (106.9 ± 14.6/min) than ostrich embryos on DD 31 (140.9 ± 15.8/min) during the first 5 min after exposure to narcotic gases (*p* < 0.001).

**FIGURE 4:**
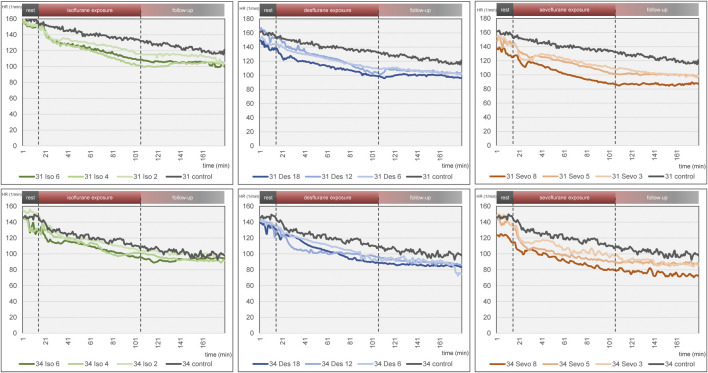
Mean heart rate over time for each narcotic gas and concentration (green: isoflurane, blue: desflurane; orange: sevoflurane). Top row: DD 31; bottom row: DD 34. In each graph, control group is depicted in grey. Dashed lines represent start and end of narcotic gas exposure. In each group, eight ostrich embryos on DD 31 and 34 were investigated, respectively.

**FIGURE 5:**
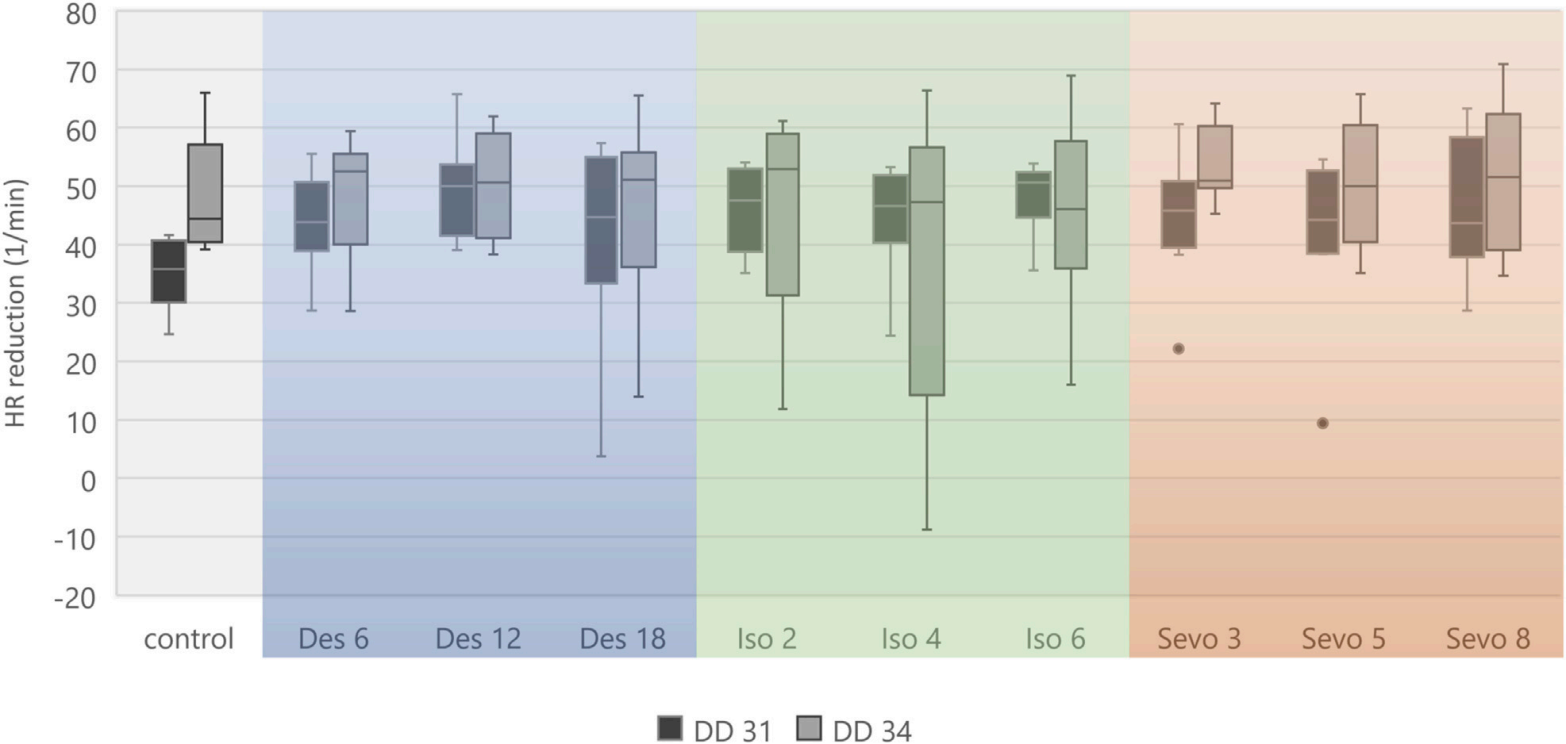
Boxplot diagram of mean heart rate reduction (difference) between 0–15 min and 151–180 min for each narcotic gas and concentration. Considering DD 31 and DD 34 separately, comparison between control group and each narcotic gas and concentration revealed no significant differences.

Figure 4 visualizes effects of isoflurane, desflurane and sevoflurane over time. Mean heart rate reduction between time intervals 0–15 min and 151–180 min was 43.8/min on DD 31 and 47.7/min on DD 34, respectively; there was no significant difference between control group and narcotic gases and different concentrations (Figure 5).

### Embryonal movement

**FIGURE 6:**
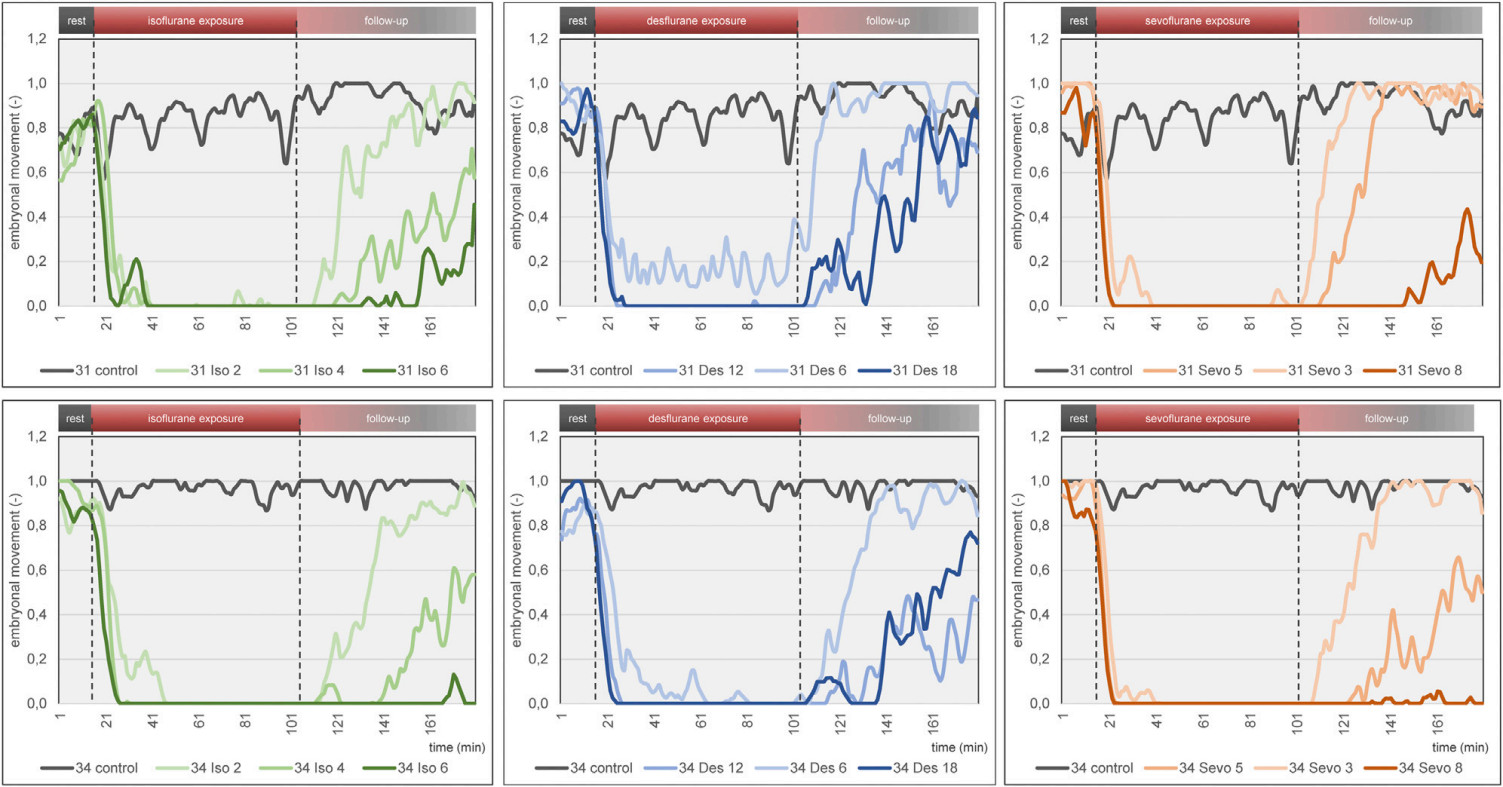
Mean embryonal movement over time for each narcotic gas and concentration (green: isoflurane, blue: desflurane; orange: sevoflurane). Top row: DD 31; bottom row: DD 34. In both rows, results of control groups are depicted in grey. Dashed lines represent start and end of narcotic gas exposure. Note the light blue graph on DD 31 and DD 34 (desflurane 6%) during narcotic gas exposure (15–105 min) which indicates residual movement whereas the other graphs (desflurane 12%, 18%; sevoflurane, isoflurane) show almost constant 0 values. This figure mainly focuses on visualization of group differences. For quantification of effectiveness of immobilization, see Figure 7.

**FIGURE 7:**
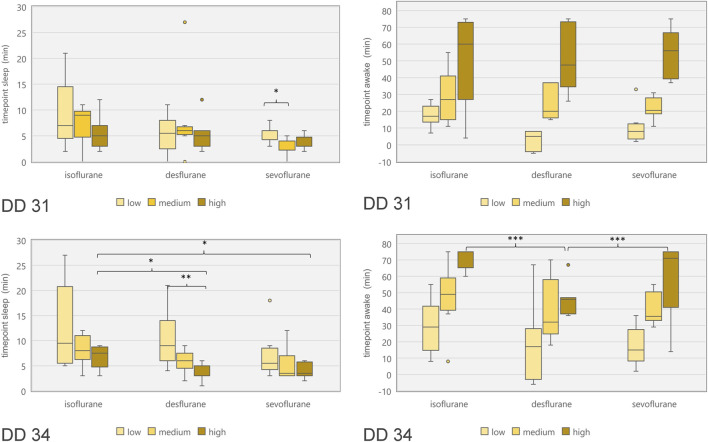
Boxplot diagram of duration between start of narcotic gas exposure and reduction of movements (“time point sleep”; left diagrams) as well as duration between end of narcotic gas exposure and re-appearance of movements (“time point awake”; right diagrams) for ostrich embryos on DD 31 (top row) and DD 34 (bottom row). In order to compare concentration levels of narcotic gases, categories “low,” “medium” and “high” were used as provided by dedicated vaporizers. Note the shorter time until re-appearance of movements for desflurane [as compared to sevoflurane and isoflurane (highest concentrations)] on DD 34. *p < 0.05; **p < 0.01; ***p < 0.001.

Embryonal movement over time is shown in Figure 6, aiming at visualization of immobilization effect. Control groups on DD 31 and DD 34 (grey lines) differ slightly without significant differences while less embryonal movement is detectable when using highest concentrations for isoflurane, desflurane and sevoflurane, respectively. Figure 7 visualizes effectiveness of immobilization using time points “sleep” and “awake” in minutes after starting and ending narcotic gas inflow, respectively. On DD 34, highest concentration of isoflurane, desflurane and sevoflurane lead to a significantly different median time point “sleep” of 7.5 ± 2.1 min, 3.0 ± 1.5 min and 3.5 ± 1.4 min, respectively (*p* = 0.0169) and a significantly different time point “awake” of 75.0 ± 5.7 min, 46.0 ± 9.6 min, and 71.0 ± 21.4 min, respectively (*p* = 0.0252). There is no significant difference between low, medium and high concentration within each narcotic gas group. On DD 31 and DD 34, sevoflurane produces a significantly shorter median time for the ostrich embryo to “sleep” (DD 31: 4.0 ± 1.7 min; DD 34: 4.0 ± 3.5 min) than isoflurane (DD 31: 7.0 ± 4.7 min; DD 34: 8.0 ± 5.5 min) and desflurane (DD 31: 6.0 ± 5.3 min; DD 34: 6.0 ± 4.5 min) (DD 31: *p* = 0.0143; DD 34: *p* = 0.0015). Over all narcotic gas groups and concentrations, ostrich embryos awake earlier on DD 31 (24.0 ± 22.1 min) than on DD 34 (37.0 ± 22.6 min) (*p* = 0.0016).

## Discussion

Isoflurane, desflurane and sevoflurane were compared in three different concentrations regarding effect on ostrich embryo motion and heart rate.

### Method agreement

Both methods of movement detection, i.e., manual movement detection and advanced automatic movement detection showed a good agreement in a dataset (comprising control group and isoflurane 6% on DD 34) retrieved from [1], thus confirming appropriateness of automatic movement detection. As data reconstruction and evaluation was shorter for the automatic approach, this method was chosen for comparison of different narcotic gases and concentrations. Initially, the algorithm for automatic movement detection was designed for human fetuses of 34–38 weeks of gestational age [26, 28]. Human fetuses of that age weigh approx. 2400–3400 g [29], which is about ten times heavier than an ostrich embryo of approx. 350 g on DD 34 and 220 g on DD 31, respectively. However, less weight did not hamper MOG signal detection as the algorithm produced adequate results. This study revealed that data reconstruction and evaluation using the automatic approach (as short as 20 min) was faster than manual movement detection which required at least 45 min for a 90 min-data file, as reported by Freesmeyer et al. [1].

### Embryonal movement

The three narcotic gases show comparable results regarding efficacy of movement reduction during exposure. After less than 8 min, ostrich embryos show a distinct cessation of movements and sevoflurane was most effective with this regard. Only one group (desflurane 6%) which represents the lowest concentration for this gas, showed residual movements during narcotic gas exposure (15–105 min) (Figure 6). After discontinuation of narcotic gas exposure, ostrich embryos having been exposed to desflurane showed a significantly shorter time until reappearance of first movements than isoflurane and sevoflurane, indicating inferior immobilization effects of desflurane (Figure 7). As expected, highest concentrations of each narcotic gas produced a longer immobilization than lowest concentration (Figure 6), however, this result was not significant (Figure 7). This finding is in line with results showing more depression of motor evoked potentials in rats being exposed to higher concentrations of isoflurane [30].

Regarding ostrich eggs, only one study has addressed the effect of immobilization using narcotic gases, i.e., isoflurane [1]; however not investigating different concentrations. Heidrich et al. have successfully used isoflurane in chicken embryos in order to enable artifact free imaging, but also did not investigate different concentrations of isoflurane [22]. Dose-dependent effects of desflurane and other narcotic gases are well-known [31] and adjustment of concentrations represent common clinical practice in anesthetic management of patients. Chambers holding ostrich eggs and preventing leakage of narcotic gases might produce image artifacts [4, 32], thus, the setup described in this study aimed at narcotic gas exposure before imaging procedure. In this context, it is very important to consider the time from the end of narcotic gas exposure and reappearance of movements. This time span is required to establish a vessel access in order to intravenously administer substances, e.g., radiopharmaceuticals for *in-vivo* imaging. On DD 34, desflurane in its highest concentration (18%) led to an earlier reappearance of movements in ostrich embryos than isoflurane and sevoflurane, respectively (Figure 7). Thus, desflurane is less suitable to suppress embryonal movement, whereas isoflurane and sevoflurane allow for artifact-free imaging after mean 70 min after the end of narcotic gas exposure.

### Heart rate

Heart rate reduction over time was present in all individuals including control group which indicates factors independent from narcotic gases as the underlying reason. Continuous cooling of ostrich eggs during experiments is considered the main factor as a mean reduction of 6.1°C was observed in this study. Similar effects were reported previously using the same methodological approach [1]. MOG requires a complex MEG-system shielding of the entire room to suppress external magnetic field changes interfering with detection of magnetic flux. Also, MEG-system relies on constant room temperature of 25°C which makes cooling of the ostrich eggs inevitable. Due to close proximity of ostrich eggs and magnetometers, thermal support with heating pads was not possible as it would have also increased the temperature of magnetometers, thus hampering MOG. Interpreting the heart rate reduction over time for each narcotic gas and concentration separately indicates a slightly pronounced decrease after initiation of narcotic gas exposure (16–20 min) and continuous (linear) reduction during narcotic gas exposure and follow-up; however, this difference is not significant (Figures 4, 5). Concluding, heart rate reduction is present in all ostrich embryos and occurs independently from narcotic gas exposure.

This study revealed low toxicity of repeated narcotic gas exposure as all individual survived experiments on DD 31 and 79/80 embryos survived experiments on DD 34. The assessment of viability 24 h after the last experiment was deemed appropriate to verify lethality. Regarding toxicity, da Rosa et al. investigated effects of isoflurane and sevoflurane in low concentrations on fertility of mice [33] and Liu et al. reported on long-term neurotoxicity of isoflurane and sevoflurane in neonatal mice [34]; however, both studies consider long-term effects and not short-term survival as in this study. Ostrich embryos were sacrificed on DD 37 at the latest, i.e., three days after last exposure to narcotic gases. Therefore, long-term effects of narcotic gases on ostrich embryos cannot be assessed using the current experimental setup.

All ostrich embryos underwent narcotic gas exposure on DD 31 and DD 34. This approach was chosen in order to show feasibility and safety of narcotic gases even in repeated experiments. As it is the goal to enable artifact-free imaging of ostrich embryos, repeated experiments in the same animal is desirable in order to reduce the number of embryos used in an experiment. Few differences were present between these two development stages which are predominantly characterized by embryo growth; organogenesis is complete at this late time point [2, 24, 35]. Namely, significant lower heart rate was observed in embryos on DD 34 in the first 5 minutes after initiation of narcotic gas exposure as well as at the end of follow-up-phase (Figure 3) and ostrich embryos on DD 31 show a faster re-appearance of movements after discontinuation of narcotic gas exposure than embryos on DD 34. These are unexpected results as the applied narcotic gas divided by embryo weight is much higher in embryos on DD 31 (data not shown) and should lead to a stronger effect than in further developed embryos. It is well known that in general, heart rate is higher in smaller and younger individuals; however, this does not explain the differences in reaction to narcotic gas exposure. One possible explanation is the repetition of narcotic gas exposure.

All embryos investigated on DD 34 had been investigated with the same narcotic gas in the same concentration 3 days before. This might indicate a cumulative effect of narcotic gases; however, this assumption cannot be proven with the current setup and would require experiments comprising a control group of embryos on DD 34 without prior narcotic gas exposure.

### Limitations

This study addresses important questions regarding the effect of narcotic gases; however, the following limitations have to be considered: The experiments investigated only two time points during development, i.e., DD 31 and 34. Especially considering unexpected results between both time points regarding heart rate, experiments on further time points, i.e., DD 37 and 28 are important to transfer feasibility of immobilization via narcotic gases.

The system setup of MOG is rather elaborate due to extensive shielding and necessary metal-free environment. More compact and mobile systems would be desirable in order to enable simultaneous detection of cardiac action/movements and acquisition of images (CT, MRI, and PET).

The concept of using ostrich embryos as an alternative for animal testing is rather new and not (yet) widely distributed and results cannot be transferred without limitations to more commonly used chicken eggs or even mammals. Furthermore, it is unknown whether narcotic gases might influence experiments (e.g., normal biodistribution of radiopharmaceuticals) in ostrich embryos.

### Conclusion

This study investigated desflurane, isoflurane and sevoflurane in three different concentrations for immobilization of ostrich embryos in order to enable motion artifact-free imaging. Minor methodological changes regarding automation of data reconstruction were successfully applied. Isoflurane and sevoflurane in their respective highest concentration (as permitted by the dedicated vaporizers), i.e. 6 % and 8%, respectively, were most suitable for immobilization which lasted for approx. 70 min after discontinuation of narcotic gas exposure. All three narcotic gases are considered safe as only one of 80 embryos (160 experiments) died after narcotic gas (i.e., desflurane) exposure and heart rate did not change significantly when compared to a control group.

## Data Availability

The raw data supporting the conclusion of this article will be made available by the authors, without undue reservation.
